# Management of Iron Overload in Infants and Toddlers With Diamond–Blackfan Anemia Syndrome: A French–Italian Study

**DOI:** 10.1002/ajh.70354

**Published:** 2026-05-11

**Authors:** Francesca Torchio, Baptiste Lecalvez, Emanuela Garelli, Coralie Mallebranche, Adriana Carando, Marie‐Pierre Castex, Nathalie Garnier, Marco Zecca, Nathalie Aladjidi, Elisa Bertoni, Arthur Sterin, Maria Licciardello, Anne Sirvent, Antonella Sau, Isabelle Marie, Benedicte Bruno, Lydie M. Da Costa, Franca Fagioli, Paola Quarello, Thierry Leblanc

**Affiliations:** ^1^ Department of Public Health and Pediatric Sciences University of Turin Turin Italy; ^2^ Department of Public Health and Pediatrics, Postgraduate School of Pediatrics University of Turin Turin Italy; ^3^ Department of Pediatric Hematology and Immunology Robert Debré Academic Hospital, GHU AP‐HP Nord Université Paris Cité Paris France; ^4^ Université Angers, Université de Nantes CHU Angers, Inserm, CNRS, CRCI2NA, SFR ICAT Angers France; ^5^ Unité D'onco‐Immuno‐Hémato Pédiatrique CHU D'Angers Angers France; ^6^ Pediatric Oncology and Hematology Department, Hôpital Des Enfants University Hospital Center of Toulouse Toulouse France; ^7^ Institut D'Hematologie et D'Oncologie Pediatrique Hospices Civils de Lyon Lyon France; ^8^ Pediatric Hematology/Oncology Fondazione IRCCS Policlinico San Matteo Pavia Italy; ^9^ Paediatric Clinical Immunology, Pellegrin Hospital, CIC1401, INSERM CICP Bordeaux University Hospital Bordeaux France; ^10^ Centre de Référence National Des Cytopénies Auto‐Immunes de L'Enfant (CEREVANCE) Bordeaux France; ^11^ Pediatric Oncohematology and Bone Marrow Transplant (BMT) Unit Children's Hospital, Spedali Civili Brescia Italy; ^12^ Department of Pediatric Hematology, Immunology and Oncology La Timone Children's Hospital Marseille France; ^13^ Pediatric Hematology and Oncology Unit, Department of Clinical and Experimental Medicine University of Catania Catania Italy; ^14^ Pediatric Haematology and Oncology Department CHU Montpellier Montpellier France; ^15^ Department of Hematology and Oncology Santo Spirito Hospital Pescara Italy; ^16^ French National Reference Center for Aplastic Anemia and PNH, CeRAMIC Saint‐Louis Hospital Paris France; ^17^ Department of Pediatric Hematology‐Oncology Jeanne de Flandre Hospital Lille France; ^18^ Paris‐Saclay University Paris France; ^19^ U1770 Inserm Gustave Roussy Institute, LABEX GR‐Ex Villejuif France; ^20^ Laboratory of Hematology Bicêtre University Hospital Le Kremlin‐Bicêtre France; ^21^ Pediatric Onco‐Hematology, Stem Cell Transplantation and Cellular Therapy Division Regina Margherita Children's Hospital Turin Italy; ^22^ Université de Paris Cité Paris France; ^23^ Inserm Unit U976 Saint‐Louis Hospital Paris France

**Keywords:** Diamond–Blackfan Anemia Syndrome, early iron chelation, iron overload

## Abstract

Diamond–Blackfan Anemia Syndrome (DBAS) is a rare congenital anemia often requiring chronic red blood cell transfusions from infancy. Without appropriate chelation, iron overload develops early and may be severe; however, no data are available on chelation in patients under 3 years of age. To address this, we conducted a retrospective, multicenter study collecting data from the French and Italian DBAS national registries. A total of 167 transfused DBAS patients were screened. Of these, 64 (38%) initiated chelation before the age of three (median: 18 months). Indications for chelation were a serum ferritin ≥ 500 ng/mL (median: 1340 ng/mL) and more than 10 transfusions. Deferasirox was the most frequently used chelator (63%), followed by deferoxamine (35%). Chelation was associated with a significant reduction in serum ferritin levels (−11% per year; *p* < 0.001). At 5–6 years of age, ferritin level was available for 28 patients: 43% had levels < 500 ng/mL, and none exceeded 2000 ng/mL. Liver iron concentration was assessed in 31/64 patients (48%) at a median age of 3.2 years; 45% showed severe overload at first evaluation. Among 22 patients who underwent cardiac magnetic resonance, no myocardial iron overload was detected. These real‐world data support the feasibility, tolerability, and effectiveness of chelation in transfusion‐dependent DBAS patients under 3 years, allowing prevention of cardiac iron overload. Although derived from DBAS, these findings may inform the management of iron overload in infants and toddlers with other transfusion‐dependent anemias and support development of age‐specific chelation strategies.

## 1 Introduction

Diamond–Blackfan Anemia Syndrome (DBAS) is a rare congenital disorder (5–10 cases per million live births) characterized by pure red cell aplasia, physical malformations, and cancer predisposition [1, 2].

Approximately 90% of patients require regular transfusions within the first year of life [3].

Although corticosteroids can be introduced after 1 year of age, around 35%–40% of children are not responsive or depend on high steroid doses, ultimately necessitating long‐term transfusion support [3].

While transfusions are life‐saving, prolonged transfusion dependence inevitably leads to chronic iron overload, which highlights iron chelation as a critical component of DBAS management.

Furthermore, compared to other transfusion‐dependent anemias, DBAS presents unique challenges.

First, transfusions often begin in the neonatal period, and the overall transfusional burden and iron load tend to be higher compared to thalassemic patients [4]. Second, DBAS patients typically exhibit elevated levels of toxic, nontransferrin‐bound iron, a phenomenon likely linked to the near absence of erythroid precursors in the bone marrow [5]. Third, despite the early onset of iron accumulation, there are currently no specific guidelines for initiating chelation therapy in infants and toddlers with DBAS, and only a recent international consensus gave some practical rules [6].

Without appropriate chelation, iron accumulates rapidly in DBAS patients, particularly in the liver, heart, pancreas, and pituitary gland, with early and severe hepatic overload frequently observed [7, 8, 9, 10].

Pancreatic and pituitary hemosiderosis is predictive of endocrinopathies, which occur in up to 53% of DBA patients and include hypogonadism, hypothyroidism, and diabetes mellitus [6, 7].

Cardiac complications are frequently documented and represent a leading cause of mortality in DBAS [4].

Over time, secondary hemochromatosis remains one of the leading causes of mortality among nontransplanted DBAS patients [11].

The 2008 DBA International clinical Consensus already emphasized the importance of chelation in transfusion‐dependent patients [1]. However, only the recently published 2024 International Consensus provided more detailed recommendations regarding the timing and monitoring of chelation therapy [6].

Currently, three chelating agents are available: deferoxamine (DFO), deferasirox (DFX), and deferiprone (DFP), the latter generally reserved for third‐line use in DBAS, due to its high risk of agranulocytosis [12].

These agents vary in administration routes, pharmacokinetics, organ‐specific efficacy, and adverse event profiles, yet all pose significant limitations in young pediatric patients [12, 13, 14, 15, 16, 17, 18, 19, 20, 21, 22, 23, 24].

In France and Italy, the marketing authorization of iron chelators places relevant therapeutic constraints on their use in infants and toddlers. DFO is approved for secondary hemosiderosis; however, in children under 3 years of age, its use requires regular growth monitoring, and the mean daily dose should not exceed 40 mg/kg.

DFX is licensed for chronic transfusion‐related iron overload in patients aged 6 years and older and may be used between 2 and 6 years when DFO is contraindicated or inadequate but with limited safety data and clinical experience. Its use below the age of two is off‐label. Moreover, only the tablet formulation is currently available in Europe, whereas in the United States a granule formulation is also available, which is significantly more suitable for young children.

DFP, on the other hand, is only approved for iron overload in thalassemia syndromes and exclusively as second‐line therapy when current chelation is ineffective or contraindicated.

Although the EMA label indicates limited data on DFP use in children under 10 years of age, recent clinical trials in transfusion‐dependent hemoglobinopathies have demonstrated efficacy and safety of DFP in younger children, including infants [24]. However, these data cannot be directly extrapolated to DBAS, where DFP use is generally discouraged due to a significantly increased risk of agranulocytosis, estimated to be up to tenfold higher compared to other populations [25].

These regulatory constraints further complicate chelation decisions for the youngest DBAS patients, often necessitating off‐label use.

Despite the clinical relevance, no studies to date have described real‐world chelation practices in DBAS patients under the age of three.

To address this gap, we conducted a retrospective, multicenter study involving patients from the French and Italian DBAS cohorts. The primary objective was to determine the proportion of transfusion‐dependent patients initiating iron chelation therapy before the age of three. Secondary objectives were to identify clinical and biological triggers for initiating chelation, to evaluate chelator selection (including type, administration routes, and dosing), to assess the effectiveness of chelation therapy in reducing iron overload, and to document adverse events.

## 2 Material and Methods

### 2.1 Study Design and Population

We conducted a retrospective, multicenter, noninterventional study based on clinical data collected from the French and Italian national DBAS registries. These are observational, longitudinal registries of pediatric and adult patients with DBAS, which prospectively collect clinical, biological, radiological, and genetic information from diagnosis. In cases where data were lacking, additional information was collected for this study using an electronic case report sent to local physicians.

Inclusion criteria were:

- Diagnosis of DBAS according to the 2024 International Consensus Statement [6].
- Birth between January 1st 2008 and December 31st 2022.
- Receipt of at least one red blood cell transfusion before 3 years of age.

The 2008 cut‐off was chosen to exclude historical records with incomplete data, while the 2022 limit ensured a minimum follow‐up period for the most recent patients.

Patients who received regular transfusions outside France or Italy during their first 3 years of life were excluded due to the limited availability of clinical data.

Initiation of iron chelation before the age of 3 years was not included among the eligibility criteria to capture the proportion of transfused patients who may require early chelation. Subsequent analyses focused on the subgroup of patients who initiated chelation therapy before the age of three.

### 2.2 Data Collection and Statistics

Data collection was completed on February 1, 2025.

All patients included in the study were diagnosed with Diamond–Blackfan anemia before the age of 3 years and had received at least one red blood cell transfusion before 3 years of age.

For the overall cohort, demographic and genetic characteristics were collected, including sex, date of birth, country and center of follow‐up, and molecular diagnosis when available.

Initiation and timing of iron chelation therapy were recorded for all patients.

For the selected population of patients who initiated chelation before 3 years of age, additional transfusion and chelation‐related data were collected.

Transfusion history included the date of the first transfusion, any history of intrauterine transfusions, and the annual frequency of transfusions.

Chelation‐related variables included the laboratory, clinical or radiological criteria used to initiate chelation (serum ferritin levels > 500 ng/mL and/or a history of more than 10 transfusions and/or evidence of tissue iron overload), the peak ferritin level recorded prior to starting chelation, the first chelating agent prescribed and any change in chelation treatment before the age of three, any treatment interruption due to reduced compliance or suspected toxicity before the age of three.

Regarding chelation prescription, we investigated the type of chelator, the starting dose, the maximum dose, and the route of administration. Any changes in chelator prescription made before the age of three were also recorded.

Over the years, various formulations of DFX have been employed. According to the product label, the recommended dose for film‐coated tablets is approximately 30% lower than that for dispersible tablets, due to increased bioavailability. Therefore, when analyzing mean prescribed doses, a correction factor was applied to prescriptions involving the dispersible formulation to ensure standardization across formulations. The dosage reported and analyzed in this study, therefore, corresponds to that of the film‐coated tablet formulation.

The effectiveness of chelation over time was primarily assessed through longitudinal analysis of serial ferritin measurements. In addition, we evaluated clinically relevant timepoints, including the last available ferritin value during ongoing chelation and ferritin levels measured between 5 and 6 years of age.

Instrumental assessments of iron overload were also reviewed, and changes in liver iron concentration (LIC) over time were analyzed.

Depending on the participating center, iron overload assessment was performed either by MRI (magnetic resonance imaging) or by SQUID (superconducting quantum interference device).

Variability in imaging modality and follow‐up intervals reflected real‐world constraints inherent to the evaluation of very young children, particularly the need for sedation and the limited feasibility of repeated examinations.

Given the different methodology used, liver iron overload was classified into five severity grades as follows: normal (LIC MRI ≤ 1.5 mg/g; LIC SQUID ≤ 0.4 mg/g wet weight), very mild (LIC MRI > 1.5 ≤ 3 mg/g; LIC SQUID > 0.4 ≤ 0.8 mg/g wet weight), mild (LIC MRI > 3 ≤ 7 mg/g; LIC SQUID > 0.8 ≤ 1 mg/g wet weight), moderate (LIC MRI > 7 ≤ 15 mg/g; LIC SQUID > 1 ≤ 2 mg/g wet weight) and severe iron overload (LIC MRI > 15 mg/g; LIC SQUID > 2 mg/g wet weight). Cardiac iron overload was defined as a T2* level < 20 ms.

Adverse events were analyzed according to exposure to each chelator before 3 years of age, irrespective of treatment sequence. Accordingly, denominators reflect the number of patients exposed to each chelator, and patients could contribute to more than one treatment group.

All adverse events were defined and graded according to the Common Terminology Criteria for Adverse Events (CTCAE) v5.0.

For long‐term outcome assessment, follow‐up data were collected using a predefined hierarchical approach from the time of chelation initiation: 10‐year data were used when available; otherwise, 5‐year data were considered; if neither was available, the last follow‐up visit during ongoing transfusion dependence and chelation therapy was used.

Continuous variables were summarized as medians with interquartile ranges (IQR) or means with ranges, as appropriate, while categorical variables were reported as counts and percentages.

Between‐group comparisons were performed using nonparametric methods (Mann–Whitney *U* test).

The association between age at chelation initiation and chelator choice was evaluated using logistic regression, with age and country included as covariates; results are reported as odds ratios with 95% confidence intervals. The deferiprone group (*n* = 1) was excluded from statistical analyses due to insufficient sample size for meaningful comparison.

Longitudinal ferritin changes were analyzed using mixed‐effects models accounting for repeated measures, as detailed in the Supplementary Methods. A two‐sided *p* < 0.05 was considered statistically significant.

All analyses were performed using Jamovi (version 2.4.8; The Jamovi Project, Sydney, Australia), an open‐source statistical platform built on R.

This study was conducted in accordance with the Declaration of Helsinki. The Italian cohort was included in the Multicenter Retrospective–Prospective Observational Study on Diamond–Blackfan Anemia, approved by the Ethics Committee of AOU Città della Salute e della Scienza di Torino, Turin, Italy (protocol number 0105777/2016; last amendment 0079681/2022). The French cohort was derived from the OFABD registry (Observatoire Français de l'Anémie de Blackfan–Diamond), registered with the French national data protection authority (CNIL, 1396823 V0).

Ethical approval for the French and Italian DBA registries includes permission for the use of recorded data in research. Consequently, no additional informed consent was required for this retrospective analysis.

## 3 Results

### 3.1 Patients

We identified 167 patients enrolled in the French (92/167; 55%) or the Italian (75/167; 45%) national DBAS registries, born between 2008 and 2022, and who received at least one transfusion before the age of three. A total of 114 patients had a confirmed genetic diagnosis (114/167; 68%) (Table S1).

Sixty‐four patients (64/167; 38%) started chelation before 3 years old: 40 from France (43% of included patients) and 24 from Italy (32%). By the time of data cutoff (February 1, 2025), the chelated cohort had a median age of 9.7 years (range, 2.1–17 years) (Table S2).

The median age at chelation initiation was 18 months (range: 4–35 months).

In all patients, the criteria for initiating chelation therapy were a serum ferritin level above 500 ng/mL and more than 10 transfusions.

Exact pre‐chelation ferritin values were available for 60 out of the 64 patients; for the remaining four patients, only the information that ferritin levels were above 500 ng/mL was recorded.

The median ferritin level prior to chelation initiation was 1340 ng/mL (IQR, 1000–1750 ng/mL).

Quantification of iron by MRI or SQUID was never used as a criterion for initiating chelation therapy.

### 3.2 Chelation Prescription

First‐choice chelators in patients under 3 years of age were as follows: DFX in 40 patients (63%), DFO in 23 patients (35%), and DFP in one patient.

The age at initiation, starting, and maximum doses are summarized in Table 1. Intravenous DFO was limited to two specific cases: one with short 15‐min infusions (an administration schedule currently not recommended) and the other with continuous infusion (24/24 h). Regarding the DFO dose, the current consensus suggests that the maximal dose under 3 years should not exceed 30 mg/kg/day in order not to impair growth [6]. Nine (9/20; 45%) of our patients received a daily dose ≤ 30 mg/kg/day. Eleven patients (11/20; 55%) received higher doses, but all remained ≤ 40 mg/kg/day which is the dose recommended in the summary of product characteristics, and only one patient was treated with 50 mg/kg/day of DFO.

**TABLE 1:** Chelators prescription.

Chelator *N* (%)	Age at initiation (months)	Starting dose (mg/kg/day)	Maximal dose^c^ (mg/kg/day)	Route of administration	Days per week
Deferoxamine^a^ 23 (35)	Median: 14	Median: 28.5	Median: 30.5	SC (*N* = 21) IV (*N* = 2)	5–7
	Mean: 14.5	Mean: 29.4	Mean: 32.9		
	Range: 4–22	Range: 20–50	Range: 20–50		
Deferasirox^b^, ^d^ 40 (63)	Median: 20.5	Median: 14	Median: 23	OS	2–7
	Mean: 21	Mean: 15	Mean: 22.7		
	Range: 6–35	Range: 7–32	Range: 8–40		
Deferiprone 1 (2)	29	75	75	OS	7

Abbreviations: IV, intravenous; SC, subcutaneous.

^a^ Data available for 20/23 patients.

^b^ Data available for 38/40 patients.

^c^ Maximal dose is for children < 3 years.

^d^ The dosage is reported in film‐coated tablets equivalents.

For DFX, there is no specific guideline available for infants or toddlers. To note, the median given dose in this cohort is rather low, and this may reflect the caution of prescribers.

Overall, 25 patients (39%) underwent changes in chelation therapy before the age of three, including switches between chelators or initiation of a combination of chelators.

A total of 11 patients (11/64; 17%) received combined chelation with DFO and DFX before the age of three. Of these, 8 were initially treated with DFO with subsequent addition of DFX, while three were initially treated with DFX with subsequent addition of DFO. Combination therapy was introduced at the discretion of the treating center, typically in the context of high iron burden or suboptimal response to monotherapy. Eight patients continued combination therapy beyond 3 years of age.

Patients treated with DFX initiated chelation at a significantly older age than those receiving deferoxamine (median 20.5 vs. 14 months, Mann–Whitney *U* test *p* < 0.001) (Table 2).

**TABLE 2:** Age at initiation of iron chelation therapy by chelator.

Variable	Patients (*n* = 63)	Deferoxamine (*n* = 23)	Deferasirox (*n* = 40)	*p*
Age at chelation initiation, months, median (IQR)	18 (4–35)	14 (11–17)	20.5 (19–28)	< 0.001^a^
France, *n* (%)	39 (62)	18 (78)	21 (52)	
Italy, *n* (%)	24 (38)	5 (22)	19 (48)	

^a^ Mann–Whitney *U* test.

This difference was consistently observed in both France (median 18 vs. 14 months) and Italy (22 vs. 14 months) (Table S3).

When age at chelation initiation was compared between countries, Italian patients tended to start chelation at a slightly older age than French patients (median 20.5 vs. 16.0 months), with a borderline statistical significance (Mann–Whitney test, *p* = 0.043) (Table S4).

In univariable logistic regression, age at chelation initiation was strongly associated with chelator choice, with each additional month increasing the odds of deferasirox use by 17% (OR 1.17, 95% CI 1.06–1.30; *p* = 0.002; McFadden *R* ^2^ = 0.168). This association remained significant after adjustment for country (OR 1.16 per month, 95% CI 1.05–1.28; *p* = 0.004), whereas country was not independently associated with chelator choice (Italy vs. France OR 2.31, 95% CI 0.64–8.34; *p* = 0.20) (Table S5).

### 3.3 Chelation Efficacy

Serum ferritin levels during follow‐up were available for 55 of the 64 patients (86%).

**FIGURE 1:**
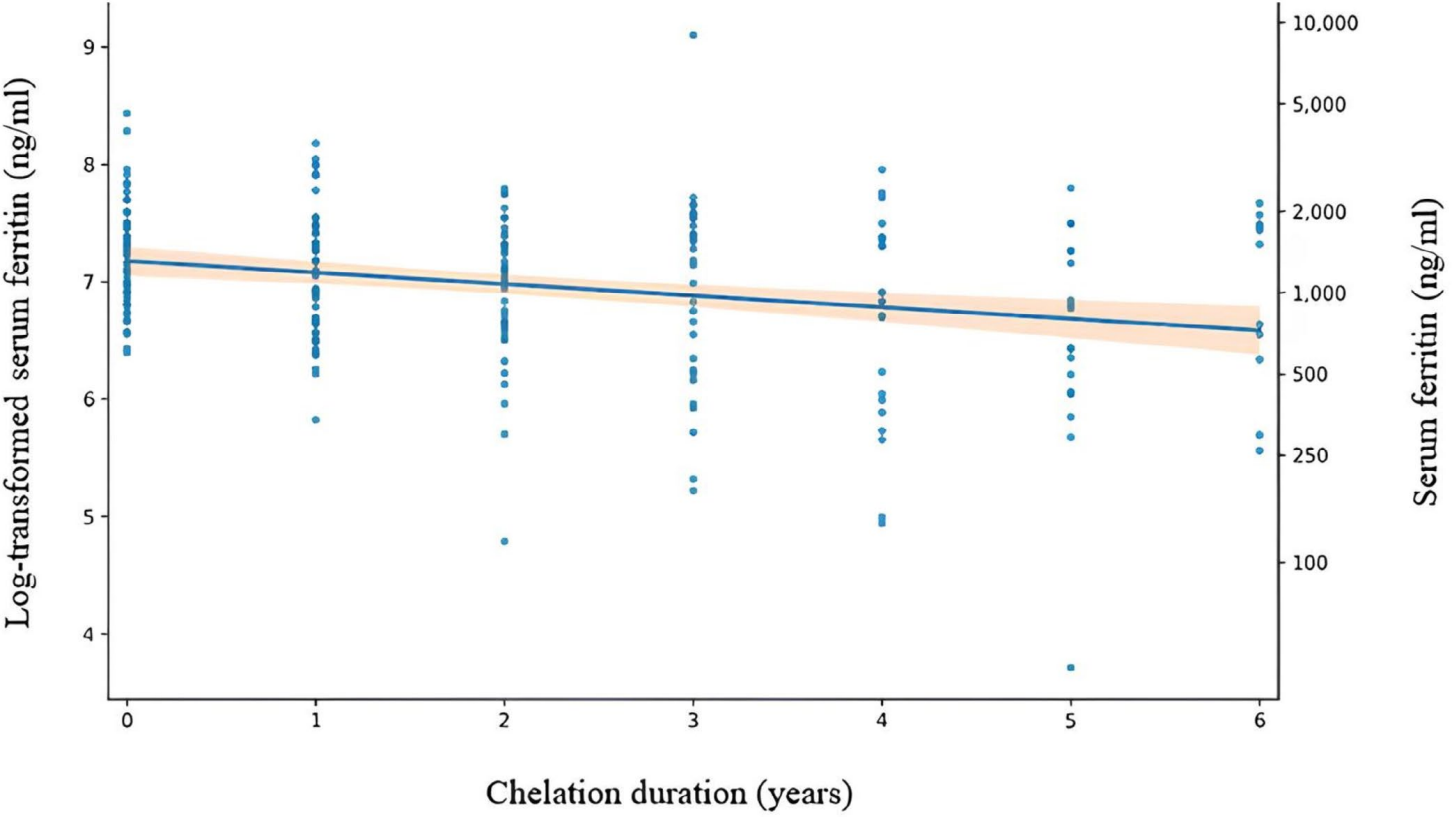
Longitudinal evolution of serum ferritin during chelation therapy. Observed log‐transformed serum ferritin values are shown as individual data points. The solid line represents fitted values from longitudinal analysis, with the shaded area indicating the 95% confidence interval. Ferritin values are shown on a natural logarithmic scale (ln). For example, ln(500) = 6.2, ln(1000) = 6.9, and ln(2000) = 7.6. [Color figure can be viewed at wileyonlinelibrary.com]

In a longitudinal mixed‐effects analysis, serum ferritin levels decreased significantly over time during chelation therapy (*β* = −0.115 per year on the log scale, 95% CI −0.155 to −0.075; *p* < 0.001), corresponding to an average annual reduction of approximately 11% (Figure 1). Additional data on serum ferritin levels over time are available in Tables S6 and S7.

A descriptive subgroup analysis according to total duration of chelation similarly showed greater ferritin reduction with longer treatment exposure (Figure S1).

In addition to the longitudinal trends in ferritin reduction, we analyzed ferritin values at a mid‐childhood follow‐up time point (5–6 years of age).

Data were available for 28 out of the 64 patients who had initiated iron chelation before the age of three. Among those, 12 patients (12/28; 43%) had serum ferritin levels below 500 ng/mL, 10 patients (10/28; 36%) had levels between 500 and 1000 ng/mL, and 6 patients (6/28; 21%) showed values between 1000 and 2000 ng/mL. Notably, no patients in this subgroup had ferritin levels exceeding 2000 ng/mL at this time point.

**FIGURE 2:**
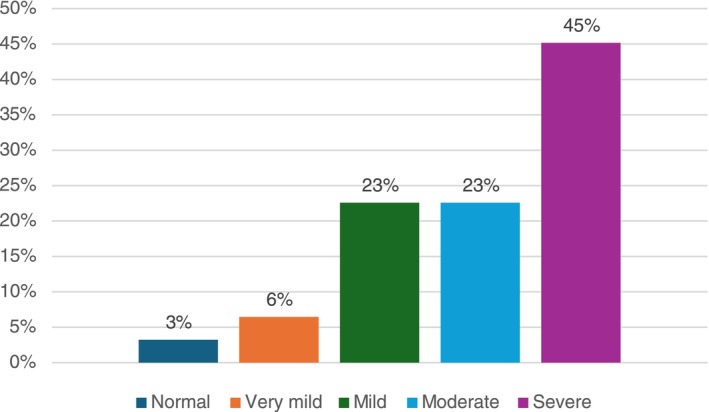
Distribution of liver iron concentration (LIC) categories at first available MRI or SQUID evaluation in transfusion‐dependent DBAS patients initiating chelation before 3 years of age. Bar chart showing the proportion of patients by LIC category: normal (LIC MRI ≤ 1.5 mg/g; LIC SQUID ≤ 0.4 mg/g wet weight; 3%, *n* = 1), very mild (LIC MRI > 1.5 ≤ 3 mg/g; LIC SQUID > 0.4 ≤ 0.8 mg/g wet weight; 6%, *n* = 2), mild (LIC MRI > 3 ≤ 7 mg/g; LIC SQUID > 0.8 ≤ 1 mg/g wet weight; 23%, *n* = 7), moderate (LIC MRI > 7–15 mg/g; LIC SQUID > 1 ≤ 2 mg/g wet weight; 23%, *n* = 7), and severe (LIC MRI > 15 mg/g; LIC SQUID > 2 mg/g wet weight; 45%, *n* = 14). MRI assessment was not systematically performed at chelation initiation and was available only in a subset of patients, reflecting real‐world practice in very young children. [Color figure can be viewed at wileyonlinelibrary.com]

Instrumental assessment of liver iron overload by MRI or SQUID was performed in 48% (31/64) of patients. The median age at first evaluation was 3.2 years (IQR, 2.8–5.8), with a mean age of 4.5 years. Among the 31 patients who underwent liver iron assessment, 14 (45%) showed severe iron overload at the time of the first evaluation (Figure 2).

All patients with severe LIC were younger than 5 years at first MRI/SQUID assessment (14/14). No statistically significant differences were observed between patients with severe and lower LIC with respect to age at transfusion initiation, age at chelation initiation, or peak serum ferritin levels prior to chelation.

Follow‐up LIC measurements were available in 18 of the 31 patients (58%), all of whom demonstrated a reduction in LIC over time. Nevertheless, two patients remained classified as having severe LIC at the last follow‐up. Both started transfusions before 1 month of age and initiated chelation before 1 year of age. However, both experienced repeated interruptions in chelation during the first 3 years of life. In the first case, subcutaneous DFO was poorly tolerated due to lower limb pain during infusion, and in both cases, DFX was discontinued due to transaminase elevation (grade 3 and grade 4, respectively). Notably, in both patients, the last MRI evaluation was performed before the age of five, and no further radiological evaluation of iron overload was available.

Cardiac MRI was performed in 22 out of 64 patients (34%) who started chelation before 3 years of age. The median age at first cardiac MRI was 4.2 years (IQR, 2.7–6.8). No patient demonstrated myocardial iron overload.

### 3.4 Chelation Discontinuation

Chelation therapy was discontinued before the age of three in 31% of patients (20/64).

Overall, 24 discontinuation episodes were recorded, as some patients experienced more than one treatment interruption due to sequential exposure to different chelators.

Discontinuation was attributed to poor compliance in 4/24 episodes (17%) and to presumed treatment‐related toxicity in 20/24 episodes (83%).

Of the 24 discontinuation episodes, 15 (63%) were temporary, eight (33%) remained permanent through 3 years of age, and in 1 case (4%) the pattern could not be determined due to loss to follow‐up. Notably, chelation was re‐initiated after 3 years of age in five cases initially classified as permanent discontinuations during the under‐3‐year study period, without recurrence of toxicity.

Adverse events were analyzed according to exposure to each chelator before 3 years of age.

Among patients treated with DFO, four discontinuations were recorded (4/23; 17%), all of which were temporary.

Among patients exposed to DFX, 20 discontinuation episodes were recorded.

Of these, 3 (3/50; 6%) were attributed to poor compliance and 17 (17/50; 34%) to presumed adverse effects, most commonly hypertransaminasemia (16/50; 32%). An additional episode of neutropenia (1/50; 2%) led to treatment discontinuation.

Among the 16 patients who experienced hypertransaminasemia, most had mild to moderate elevations in liver enzymes. According to CTCAE v5.0, cases were classified as follows: grade 1, 1/16 (7%); grade 2, 4/16 (25%); grade 3, 7/16 (44%); and grade 4, 2/16 (12%), while grading was not specified in two cases.

Higher‐grade toxicities were more frequently associated with treatment discontinuation before 3 years of age. Liver toxicity was limited to hypertransaminasemia, and no cases of liver failure were observed.

Detailed information on discontinuation patterns, including causes, severity, and treatment re‐initiation, is provided in Table S8.

### 3.5 Follow‐Up

Follow‐up data were available for 63/64 patients. At the predefined follow‐up assessment (median age 6 years, range 1–15), 36 patients remained transfusion‐dependent, 22 had undergone HSCT, and 5 had achieved treatment independence. Overall, 37 patients remained on iron chelation therapy (Table S9).

## 4 Discussion

This study represents the first multicenter effort to describe real‐life iron chelation practices in transfusion‐dependent DBAS patients under the age of three, a uniquely vulnerable population for which evidence is extremely limited.

Young DBAS patients pose a unique challenge regarding chelation, as they are particularly prone to early and severe iron overload.

Physicians should be aware that the first years of life represent an especially critical period for transfusion‐dependent patients due to the ratio of transfused iron to patient weight. For example, a 10 kg‐child receiving 200 mL of RBC per month accumulates approximately 2.4 g of iron annually (240 mg/kg/year), nearly twice the per‐kilogram dose of a 70 kg adult monthly transfused with three RBC units of 250 mL (129 mg/kg/year).

Our findings confirm the clinical relevance of early chelation in DBAS and support its growing integration into current clinical practice. A notable finding is that one in three transfused DBAS patients was put on chelation therapy before the age of three, with a median age at initiation of 18 months.

We cannot exclude the possibility that the proportion of patients who should receive a chelation is actually higher, but this is remarkably earlier than what has been historically reported in other transfusion‐dependent anemias, such as β‐thalassemia. A recent publication on thalassemic patients highlighted the scarcity of data on chelation therapy in patients under the age of two [25]. In this context, the DBAS population, due to their very early transfusion dependence and more severe iron loading than other patients with transfusion support, represents a unique challenge and a valuable model for studying early‐onset iron overload and the associated chelation strategies.

Our results on the real‐life initiation of chelation align with the recommendations of the recent 2024 International Consensus on DBAS, which highlights the importance of early chelation [6].

The consensus defines specific criteria for initiating therapy, including the accumulation of at least 10 transfusions or biochemical evidence of iron overload (e.g., transferrin saturation > 60%, serum ferritin > 500 ng/mL, or elevated LIC), while suggesting a delay in infants without severe iron overload until the first steroid trial has been completed. However, in our cohort, the mean serum ferritin level at chelation initiation was approximately 1300 ng/mL, indicating that treatment often began later than currently recommended. This delay may reflect concerns regarding the safety of chelation in very young children and the off‐label use of currently available chelators in this age group. Nonetheless, chelation was initiated in 11 infants in our study, highlighting the growing concern among physicians about early control of iron overload.

The choice of chelating agent in our cohort was consistent with international recommendations. The median age at chelation initiation was lower in patients receiving DFO than in those treated with DFX, reflecting a tendency to use DFO in younger children. Only one patient received DFP, a drug generally reserved for third‐line use and not considered a first‐line agent in DBAS due to its safety profile [12, 26]. Of note, 11 patients (17%) received a combination of two chelators before the age of three, highlighting the severity of iron burden in some patients and the clinical need for early, intensified therapeutic strategies.

Both DFO and DFX appear to be suitable. Notably, the choice of one chelator over another may also be influenced by parents' preferences, patient's compliance and the availability of delivering DFO subcutaneously via a 10–12 h infusion. Doses and administration routes were largely in line with current recommendations.

In terms of efficacy, our longitudinal analyses demonstrate a significant and sustained reduction in serum ferritin over time during chelation therapy, corresponding to an average annual decrease of approximately 11%. Importantly, this effect was observed despite ongoing transfusion dependence, supporting the capacity of early and structured chelation to progressively mitigate iron burden.

Instrumental monitoring of liver iron overload was performed in 48% of patients, with a reassuringly high proportion undergoing MRI or SQUID evaluation despite the need for sedation. Notably, all patients presenting with severe LIC were under the age of five, highlighting the importance of early imaging when possible. As also stated in the 2024 DBAS consensus, LIC should be assessed no later than the age of five, or earlier if clinically indicated, even if sedation is required [6]. Importantly, our data showed no clear correlation between serum ferritin levels and severe LIC, reinforcing the well‐known concept that ferritin alone is not a reliable marker to precisely quantify the level of iron overload and should be supplemented by MRI imaging when possible.

These imaging findings compare favorably with previous data reported by Berdoukas et al., who evaluated tissue iron burden in chronically transfused children, including 17 DBA patients [8]. In that study, cardiac iron overload was detected in 19%, including DBAS children as young as 2 years. By contrast, in our cohort, none of the patients who initiated chelation before the age of three and underwent cardiac MRI showed myocardial iron overload. These differences suggest that early initiation of iron chelation in young children with DBAS may prevent cardiac iron overload.

Evaluating the safety profile of chelation in this age group proved to be challenging, especially for DFX. Hypertransaminasemia was observed in 32% (16/50) of patients treated with DFX and was the leading cause of treatment discontinuation, as this drug is known to induce rare but severe liver toxicity.

However, it is important to note that establishing a direct causal relationship was not possible in this retrospective setting due to several confounding factors. Elevated liver enzymes may reflect underlying iron overload (as in one patient with grade 4 enzyme elevation and markedly elevated LIC) or concomitant viral infections, which are common in pediatric patients. In one case, a family history of transaminase elevation was also reported.

We cannot exclude a specific pattern of liver toxicity in children under 3 years, but despite its frequency, hypertransaminasemia was not associated with serious clinical outcomes: no liver failure or drug‐related deaths occurred, and no patients required admission to the intensive care unit, suggesting a generally mild and reversible course. Furthermore, DFX was resumed under 3 years of age in four of the seven patients who had experienced grade 3 hypertransaminasemia. For grade 4 toxicity, data were limited to a single patient, in whom the physician opted to switch chelation therapy.

No cases of severe renal toxicity were documented; however, it is worth noting that key markers of early tubular dysfunction (e.g., serum phosphate, urinary protein markers) were not systematically collected, which limit the sensitivity of our safety evaluation.

This study has several limitations. As DBAS is a rare disease, the sample size is relatively small. Additionally, its retrospective design and reliance on registry data from two national databases meant that complete information was not available for all patients. This limitation particularly affected the analysis of drug‐related toxicities and the evaluation of instrumental iron overload.

We can also suspect that clinical practice varies among centers and that more recent patients may have received earlier chelation according to recent consensus.

In conclusion, our findings support the feasibility, safety, and effectiveness of early iron chelation in transfusion‐dependent DBAS patients under 3 years of age. Early chelation seems to be associated with a significant reduction of severe liver iron loading and to prevent cardiac iron overload. Our findings underscore the need for earlier intervention, more systematic monitoring, and age‐adapted therapeutic strategies.

These real‐world data can support clinical practice and provide a foundation for future prospective studies aimed at optimizing chelation therapy in the youngest transfused DBAS patient and in other transfusion dependent anemias.

## Author Contributions

F.T., P.Q., and T.L. designed and conducted the study, analyzed and interpreted the data, and wrote the manuscript. All other authors contributed to data acquisition and reviewed the manuscript. All authors approved the final version.

## Funding

The authors have nothing to report.

## Conflicts of Interest

The authors declare no conflicts of interest.

## Supporting information


**Table S1:** Baseline demographic, clinical, and genetic characteristics of the overall cohort (*N* = 167).
**Table S2:** Demographics, genetics, clinical and chelation‐related characteristics of patients starting chelation before 3 years of age.
**Table S3:** Distribution of age at chelation initiation stratified by chelator and country. Data are presented as median, interquartile range (IQR), and range.
**Table S4:** Nonparametric comparisons of age at chelation initiation between chelator groups and between countries using the Mann–Whitney *U* test.
**Table S5:** Logistic regression models evaluating the association between age at chelation initiation and chelator choice, before and after adjustment for country.
**Table S6:** Serum ferritin levels during chelation therapy by year of follow‐up. For each time point, the number of available ferritin measurements is reported together with the median and interquartile range (IQR). Values are shown in ng/mL. The number of observations varies across time points due to variable follow‐up duration and missing data inherent to the retrospective study design.
**Table S7:** Results of the linear mixed‐effects model evaluating longitudinal changes in serum ferritin levels during chelation therapy. Serum ferritin values were log‐transformed prior to analysis. Time since chelation initiation (years) was included as a fixed effect, with patient‐specific random intercepts to account for repeated measurements. Regression coefficients (*β*) are reported with corresponding 95% confidence intervals (CI) and *p*‐values.
**Figure S1:** Change in serum ferritin levels according to the duration of chelation therapy boxplots show the distribution of Δ serum ferritin (ng/mL; last available follow‐up minus baseline at chelation initiation) across three mutually exclusive patient groups defined by total observed chelation duration: G1 (< 3 years), G2 (3 to < 6 years), G3 (≥ 6 years). Negative values indicate a reduction in iron burden. The horizontal line indicates the median and the square the mean.
**Table S8:** Chelation discontinuation events before 3 years of age and treatment re‐initiation during follow‐up. Permanent discontinuation before age 3 indicates interruption of the chelator with no re‐initiation before the age of three. Continuation or re‐initiation during follow‐up indicates whether the same chelator was resumed at any time after discontinuation during subsequent follow‐up. Each row represents a distinct chelation discontinuation event; individual patients may contribute more than one event. DFX, deferasirox; DFO, deferoxamine; NA, not available; NS, not specified.
**Table S9:** Patient‐level clinical characteristics and iron status at last follow‐up. Serum ferritin values correspond to the most recent available measurement obtained within ±12 months of the last follow‐up visit. Treatment‐independent refers to patients requiring neither transfusion support nor steroid therapy at last follow‐up. A dash (−) indicates not applicable; NA indicates data not available. DFO, deferoxamine; DFP, deferiprone; DFX, deferasirox; HSCT, hematopoietic stem cell transplantation.

## Data Availability

The data that support the findings of this study are available from the corresponding author upon reasonable request.
