# Supplementary material for: Management of Iron Overload in Infants and Toddlers With Diamond–Blackfan Anemia Syndrome: A French–Italian Study
Source: Am J Hematol. 2026 May 11;101(8):1856–65. doi: 10.1002/ajh.70354 (PMC13331634; doi:10.1002/ajh.70354)
Supplement: Supplementary file 1 — Table S1: Baseline demographic, clinical, and genetic characteristics of the overall cohort (N = 167). Table S2: Demographics, genetics, clinical and chelation‐related characteristics of patients starting chelation before 3 years of age. Table S3: Distribution of age at chelation initiation stratified by chelator and country. Data are presented as median, interquartile range (IQR), and range. Table S4: Nonparametric comparisons of age at chelation initiation between chelator groups and between countries using the Mann–Whitney U test. Table S5: Logistic regression models evaluating the association between age at chelation initiation and chelator choice, before and after adjustment for country. Table S6: Serum ferritin levels during chelation therapy by year of follow‐up. For each time point, the number of available ferritin measurements is reported together with the median and interquartile range (IQR). Values are shown in ng/mL. The number of observations varies across time points due to variable follow‐up duration and missing data inherent to the retrospective study design. Table S7: Results of the linear mixed‐effects model evaluating longitudinal changes in serum ferritin levels during chelation therapy. Serum ferritin values were log‐transformed prior to analysis. Time since chelation initiation (years) was included as a fixed effect, with patient‐specific random intercepts to account for repeated measurements. Regression coefficients (β) are reported with corresponding 95% confidence intervals (CI) and p‐values. Figure S1: Change in serum ferritin levels according to the duration of chelation therapy boxplots show the distribution of Δ serum ferritin (ng/mL; last available follow‐up minus baseline at chelation initiation) across three mutually exclusive patient groups defined by total observed chelation duration: G1 (< 3 years), G2 (3 to < 6 years), G3 (≥ 6 years). Negative values indicate a reduction in iron burden. The horizontal line indicates the median and [file AJH-101-1856-s001.docx]

***Supplementary Material and Methods***

***Supplementary Methods***

Longitudinal changes in serum ferritin levels were analyzed using a linear mixed-effects model to account for repeated measurements within individuals. Serum ferritin values were log-transformed prior to analysis to reduce skewness. Time since chelation initiation (years) was included as a fixed effect, and patient-specific random intercepts were used. Model estimates are reported as regression coefficients with corresponding 95% confidence intervals and p-values.

***Supplemenary Results***

| **Demographic/Clinical/Genetic Characteristics** | **All patients (N=167)** |
| --- | --- |
| DBA diagnosis < 3 years, n (%) | 167 (100) |
| Transfusion < 3 years, n (%) | 167 (100) |
| **Transfusion independence < 3 years, n (%)** |  |
| YES | 73(44) |
| HSCT | 15 |
| Steroid response | 40 |
| Treatment independence | 18 |
| NO | 94(56) |
| **Start of chelation < 3 years, n (%)** |  |
| < 3 years | 64 (38) |
| > 3 years or no chelation start | 103 (62) |
| **Country, n (%)** |  |
| France | 92 (55) |
| Italy | 75 (45) |
| **Sex, n (%)** |  |
| Male | 78 (47) |
| Female | 89 (53) |
| **Genetics, n (%)** |  |
| Gene | 114 (68) |
| *RPL11* | 6 |
| *RPL15* | 1 |
| *RPL17* | 2 |
| *RPL35A* | 6 |
| *RPL5* | 19 |
| *RPS10* | 1 |
| *RPS17* | 2 |
| *RPS19* | 54 |
| *RPS24* | 1 |
| *RPS26* | 20 |
| *RPS7* | 1 |
| *TP53* | 1 |
| No gene identified | 53 (32) |

**Table S1**. **Baseline demographic, clinical, and genetic characteristics of the overall cohort (N=167).**

| **Demographic/Clinical/Genetic Characteristics** | **Selected cohort (N=64)** |
| --- | --- |
| **Country, n (%)** |  |
| France | 40 (62) |
| Italy | 24 (38) |
| **Sex, n (%)** |  |
| Male | 30 (47) |
| Female | 34 (53) |
| **Genetics, n (%)** |  |
| Gene | 41 (64) |
| *RPL11* | 1 |
| *RPL15* | 0 |
| *RPL17* | 1 |
| *RPL35A* | 4 |
| *RPL5* | 4 |
| *RPS19* | 23 |
| *RPS24* | 1 |
| *RPS26* | 7 |
| No gene identified | 23 (36) |
| **Transfusions** |  |
| Transfusione < 3 years, n (%) | 64(100) |
| Age at first transfusion, days | 38 (0-69); n = 61 |
| Intrauterine transfusions, n (%) | 1 (2) |
| Number of transfusions/year < 3 years, n (%) |  |
| < 10 transfusions/year | 4 (6) |
| 10 - 20 transfusions/year | 57 (89) |
| > 20 transfusions/year | 3 (5) |
| **Chelation** |  |
| Age at chelation initiation, months | 18 (4-35) |
| Ferritin > 500 ng/ml | 64 (100) |
| Ferritin pre-chelation, ng/ml | 1,340 (1,000–1,750); n=60/64 |
| **State of patients at data cut-off** |  |
| Alive, n (%) | 63 (98) |
| Lost to follow-up, n (%) | 1(2) |
| Age of patients at data cut-off, years | 9.7 (2.1-17.0) |

**Table S2**. **Demographics, genetics, clinical and chelation-related characteristics of patients starting chelation before 3 years of age.**

**Legend.**Values are reported as median (interquartile range) or number (percentage), as appropriate.

| **Country** | **Chelator** | **N** | **Age at chelation initiation, months** | **IQR (25th–75th)** | **Range** |
| --- | --- | --- | --- | --- | --- |
| France | Deferoxamine | 18 | 14 | 11–16.8 | 4–22 |
| France | Deferasirox | 21 | 18 | 14–25 | 9–35 |
| Italy | Deferoxamine | 5 | 14 | 14–20 | 10–22 |
| Italy | Deferasirox | 19 | 22 | 19–28.5 | 6–34 |

**Table S3. Distribution of age at chelation initiation stratified by chelator and country. Data are presented as median, interquartile range (IQR), and range.**

| **Comparison** | **N** | **Median (months)** | **Test** | **Statistic** | **p-value** |
| --- | --- | --- | --- | --- | --- |
| Deferasirox vs Deferoxamine | 40 vs 23 | 20.5 vs 14.0 | Mann–Whitney | U = 226 | < 0.001 |
| Italy vs France | 24 vs 39 | 20.5 vs 16.0 | Mann–Whitney | U = 325 | 0.043 |

**Table S4. Non-parametric comparisons of age at chelation initiation between chelator groups and between countries using the Mann–Whitney U test.**

| **Model** | **Predictor** | **OR** | **95% CI** | **p** |
| --- | --- | --- | --- | --- |
| Univariable | Age at chelation initiation (per month) | 1.17 | 1.06–1.30 | 0.002 |
| Multivariable | Age at chelation initiation (per month) | 1.16 | 1.05–1.28 | 0.004 |
|  | Italy vs France | 2.31 | 0.64–8.34 | 0.20 |

**Table S5. Logistic regression models evaluating the association between age at chelation initiation and chelator choice, before and after adjustment for country.**

| **Time since chelation start (years)** | **n observations** | **Median ferritin (ng/mL)** | **IQR (ng/mL)** |
| --- | --- | --- | --- |
| 0 | 60 | 1350 | 1000–1750 |
| 1 | 54 | 1169 | 776–1671 |
| 2 | 42 | 1206 | 780–1511 |
| 3 | 31 | 1266 | 509–1823 |
| 4 | 24 | 931 | 417–1569 |
| 5 | 19 | 640 | 463–1356 |
| 6 | 11 | 764 | 587–1771 |

**Table S6.** Serum ferritin levels during chelation therapy by year of follow-up. For each time point, the number of available ferritin measurements is reported together with the median and interquartile range (IQR). Values are shown in ng/mL. The number of observations varies across time points due to variable follow-up duration and missing data inherent to the retrospective study design.

| **Fixed effect** | **β (log scale)** | **95% CI** | **p-value** |
| --- | --- | --- | --- |
| Time since chelation initiation (per year) | −0.115 | −0.155 to −0.075 | <0.001 |

**Table S7**. Results of the linear mixed-effects model evaluating longitudinal changes in serum ferritin levels during chelation therapy. Serum ferritin values were log-transformed prior to analysis. Time since chelation initiation (years) was included as a fixed effect, with patient-specific random intercepts to account for repeated measurements. Regression coefficients (β) are reported with corresponding 95% confidence intervals (CI) and p-values.


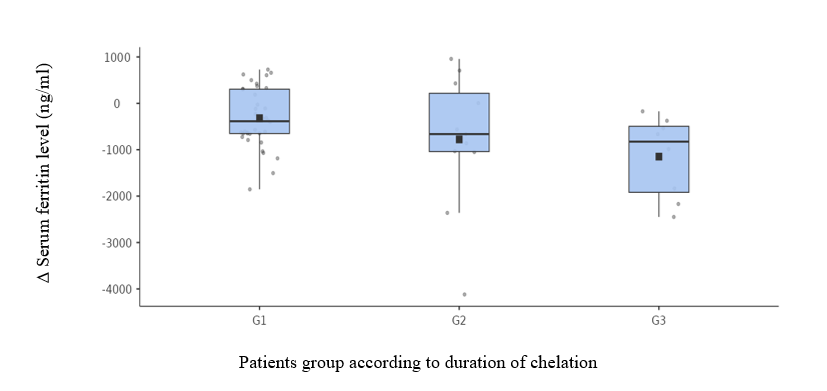


**Figure S1. Change in serum ferritin levels according to the duration of chelation therapy** Boxplots show the distribution of Δ serum ferritin (ng/mL; last available follow-up minus baseline at chelation initiation) across three mutually exclusive patient groups defined by total observed chelation duration: G1 (<3 years), G2 (3 to <6 years), G3 ( ≥6 years). Negative values indicate a reduction in iron burden. The horizontal line indicates the median and the square the mean.

Patients were stratified into three mutually exclusive groups according to total observed duration of chelation: <3 years (G1, n = 36), 3 to <6 years (G2, n = 11), and ≥6 years (G3, n = 8). The magnitude of change in serum ferritin (Δ ferritin, defined as last available follow-up minus baseline at chelation initiation) increased with longer chelation exposure. Mean Δ ferritin was −320 ng/mL in G1, −777 ng/mL in G2, and −1,149 ng/mL in G3, with corresponding median values of −386 ng/mL (IQR −651 to 305), −663 ng/mL (IQR −1,040 to 217), and −826 ng/mL (IQR −1,919 to −495), respectively (Figure S1).

| Event No. | Patient ID | Chelator | Reason for discontinuation | CTCAE grade | Permanent discontinuation before age 3 | Continuation or re-initiation of the same chelator during follow-up |
| --- | --- | --- | --- | --- | --- | --- |
| 1 | 3 | DFX | Hepatic toxicity | Grade 3 | Yes | Yes |
| 2 | 24 | DFX | Hepatic toxicity | Grade 3 | Yes | Yes |
| 3 | 25 | DFO | Pain | - | No | Yes |
| 4 | 25 | DFX | Hepatic toxicity | Grade 2 | Yes | Yes |
| 5 | 25 | DFX | Neutropenia | - | Yes | Yes |
| 6 | 5 | DFX | Poor compliance | - | No | Yes |
| 7 | 5 | DFO | Poor compliance | - | No | Yes |
| 8 | 47 | DFO | Poor compliance | - | No | Yes |
| 9 | 28 | DFX | Hepatic toxicity | NS | Yes | No |
| 10 | 48 | DFX | Poor compliance | - | No | Yes |
| 11 | 49 | DFO | Poor compliance | - | No | Yes |
| 12 | 49 | DFX | Hepatic toxicity | Grade 2 | No | Yes |
| 13 | 33 | DFX | Hepatic toxicity | Grade 3 | Yes | No |
| 14 | 34 | DFX | Hepatic toxicity | Grade 4 | Yes | No |
| 15 | 51 | DFX | Hepatic toxicity | Grade 4 | NA | NA |
| 16 | 52 | DFX | Poor compliance | - | No | Yes |
| 17 | 53 | DFX | Hepatic toxicity | Grade 2 | Yes | Yes |
| 18 | 54 | DFX | Hepatic toxicity | Grade 3 | No | Yes |
| 19 | 55 | DFX | Hepatic toxicity | Grade 2 | No | Yes |
| 20 | 56 | DFX | Hepatic toxicity | Grade 1 | No | Yes |
| 21 | 21 | DFX | Hepatic toxicity | Garde 3 | No | Yes |
| 22 | 22 | DFX | Hepatic toxicity | NS | No | No |
| 23 | 59 | DFX | Hepatic toxicity | Grade 3 | No | Yes |
| 24 | 62 | DFX | Hepatic toxicity | Grade 3 | No | Yes |

**Table S8. Chelation discontinuation events before 3 years of age and treatment re-initiation during follow-up.** Permanent discontinuation before age 3 indicates interruption of the chelator with no re-initiation before the age of three.
Continuation or re-initiation during follow-up indicates whether the same chelator was resumed at any time after discontinuation during subsequent follow-up. Each row represents a distinct chelation discontinuation event; individual patients may contribute more than one event. Abbreviations: DFX, deferasirox; DFO, deferoxamine; NS, not specified; NA, not available.

| Patient ID | Initial chelator | Age at Last Follow-up (years) | Clinical status at last follow-up | Age at HSCT (years) | On Chelation at Last Follow-up | Chelator at Last Follow-up | Last Serum Ferritin (±12 Months) (ng/mL) |
| --- | --- | --- | --- | --- | --- | --- | --- |
| 1 | DFO | 8 | Transfusion-dependent | - | YES | DFO + DFX | 285 |
| 2 | DFO | 10 | Transfusion-dependent | - | YES | DFO + DFX | 1256 |
| 3 | DFX | 9 | Post-HSCT | 5 | NO | - | 742 |
| 4 | DFX | 10 | Transfusion-dependent | - | YES | DFO | 843 |
| 5 | DFX | 10 | Post-HSCT | 3 | NO | - | < 500 |
| 6 | DFX | 10 | Post-HSCT | 9 | NO | - | 1180 |
| 7 | DFX | 10 | Treatment-independent | - | NO | - | 91 |
| 8 | DFX | 11 | Transfusion-dependent | - | YES | DFP | 928.6 |
| 9 | DFX | 11 | Transfusion-dependent | - | YES | DFO + DFP | 455 |
| 10 | DFX | 12 | Treatment-independent | - | NO | - | 185 |
| 11 | DFO | 10 | Post-HSCT | 2 | NO | - | 215 |
| 12 | DFX | 11 | Post-HSCT | 7 | NO | - | 527 |
| 13 | DFO | 13 | Post-HSCT | 2 | NO | - | 62 |
| 14 | DFO | 10 | Post-HSCT | 5 | NO | - | 200 |
| 15 | DFX | 15 | Post-HSCT | 4 | NO | - | 60 |
| 16 | DFO | 11 | Transfusion-dependent | - | YES | DFP | 697 |
| 17 | DFX | 11 | Transfusion-dependent | - | YES | DFX | 522 |
| 18 | DFX | 10 | Treatment-independent | - | NO | - | 388 |
| 19 | DFX | 14 | Transfusion-dependent | - | YES | DFX | 844 |
| 20 | DFX | 11 | Transfusion-dependent | - | YES | DFX | NA |
| 21 | DFX | 10 | Transfusion-dependent | - | YES | DFX | < 500 |
| 22 | DFX | 10 | Post-HSCT | 2 | NO | - | 288 |
| 23 | DFO | 6 | Post-HSCT | 3 | NO | - | 786 |
| 24 | DFO | 6 | Post-HSCT | 4 | NO | - | 615 |
| 25 | DFO | 3 | Transfusion-dependent |  | YES | DFO + DFX | 1182 |
| 26 | DFX | 7 | Post-HSCT | 4 | NO | - | 840 |
| 27 | DFO | 5 | Post-HSCT | 1 | NO | - | 136.6 |
| 28 | DFX | 6 | Post-HSCT | 4 | NO | - | 1117 |
| 29 | DFO | 5 | Post-HSCT | 4 | NO | - | 1749 |
| 30 | DFX | 6 | Post-HSCT | 5 | NO | - | 3134 |
| 31 | DFO | 4 | Transfusion-dependent | - | YES | DFO + DFX | 387 |
| 32 | DFX | 5 | Treatment-independent | - | NO | - | - |
| 33 | DFX | 8 | Post-HSCT | 7 | YES | DFX | 872 |
| 34 | DFX | 5 | Transfusion-dependent | - | YES | DFO | 1500 |
| 35 | DFX | 5 | Transfusion-dependent | - | YES | DFX | 1604 |
| 36 | DFX | 6 | Post-HSCT | 5 | NO | - | 1150 |
| 37 | DFX | 6 | Post-HSCT | 4 | NO | - | 1328 |
| 38 | DFO | 4 | Post-HSCT | 3 | NO | - | 1223 |
| 39 | DFX | 6 | Post-HSCT | 5 | NO | - | 821 |
| 40 | DFX | 5 | Post-HSCT | 2 | NO | - | NA |
| 41 | DFO | 3 | Transfusion-dependent | - | YES | DFO + DFX | 840 |
| 42 | DFO | 3 | Transfusion-dependent | - | YES | DFX | 850 |
| 43 | DFX | 2 | Transfusion-dependent | - | YES | DFX | 1174 |
| 44 | DFO | 2 | Transfusion-dependent | - | YES | DFO | 598 |
| 45 | DFP | 3 | Transfusion-dependent | - | YES | DFP | 984 |
| 46 | DFO | 2 | Transfusion-dependent | - | YES | DFO | 655 |
| 47 | DFO | 1 | Transfusion-dependent | - | YES | DFO | 313 |
| 48 | DFX | 3 | Transfusion-dependent | - | YES | DFX | 286 |
| 49 | DFO | 3 | Transfusion-dependent | - | YES | DFX | 1485 |
| 50 | DFX | 3 | Transfusion-dependent | - | YES | DFX | 1796 |
| 51 | DFX | NA | NA | NA | NA | NA | NA |
| 52 | DFX | 2 | Transfusion-dependent | - | YES | DFO + DFX | 1514 |
| 53 | DFX | 5 | Transfusion-dependent | - | YES | DFO | 1800 |
| 54 | DFX | 2 | Transfusion-dependent | - | YES | DFX | 977 |
| 55 | DFX | 3 | Transfusion-dependent | - | YES | DFX | 1723 |
| 56 | DFO | 3 | Transfusion-dependent | - | YES | DFO | 1500 |
| 57 | DFX | 2 | Transfusion-dependent | - | YES | DFX | 1349 |
| 58 | DFX | 7 | Transfusion-dependent | - | YES | DFX | 620 |
| 59 | DFX | 3 | Transfusion-dependent | - | YES | DFX | 3550 |
| 60 | DFO | 1 | Transfusion-dependent | - | YES | DFO | 2767 |
| 61 | DFO | 2 | Transfusion-dependent | - | YES | DFX | 500 |
| 62 | DFX | 2 | Transfusion-dependent | - | YES | DFX | 1817 |
| 63 | DFO | 5 | Treatment-independent | - | NO | - | NA |
| 64 | DFX | 2 | Transfusion-dependent | - | YES | DFX | 1137 |

**Table S9. Patient-level clinical characteristics and iron status at last follow-up.** Serum ferritin values correspond to the most recent available measurement obtained within ±12 months of the last follow-up visit. Treatment-independent refers to patients requiring neither transfusion support nor steroid therapy at last follow-up. A dash (–) indicates not applicable; NA indicates data not available. Abbreviations: DFO, deferoxamine; DFX, deferasirox; DFP, deferiprone; HSCT, hematopoietic stem cell transplantation
